# Research on the Anticorrosion Properties of CeO_2_-GO/EP Nanocomposite Coating in Simulated Sea Water

**DOI:** 10.3390/polym13132072

**Published:** 2021-06-24

**Authors:** Xiaoyan Liu, Ruidan Liu, Tianyu Li, Yanqi Liu, Li Liu, Kai Lyu, Surendra P. Shah

**Affiliations:** 1 College of Mechanics and Materials, Hohai University, Nanjing 210000, China; 201308030004@hhu.edu.cn (R.L.); 170408070004@hhu.edu.cn (T.L.); 191608010005@hhu.edu.cn (Y.L.); 191308050002@hhu.edu.cn (L.L.); 2 College of Civil and Transportation Engineering, Hohai University, Nanjing 210000, China; 3 Center for Advanced Cement-Based Materials (ACBM), Northwestern University, Evanston, IL 60208, USA; s-shah@northwestern.edu

**Keywords:** CeO_2_-GO, corrosion inhibitor, simulated seawater, corrosion area

## Abstract

Graphene is a two-dimensional sheet of regular hexagonal honeycomb lattice formed by sp^2^ hybrid orbital bonding, with only one layer thickness of a single atom, which is known as the “super king” of the 21st century. Previous studies have shown that cerium oxide-graphene oxide (CeO_2_-GO(4:1)) nanocomposites eliminated the agglomeration of graphene to some extent and the CeO_2_-GO(4:1) epoxy coating could be prepared with good anti-corrosion performance. In this paper, CeO_2_-GO(4:1) nanocomposites were prepared by the hydrothermal synthesis method, and the three-electrode method was used for electrochemical tests. The state evolution of CeO_2_-GO(4:1)/EP coating and the synergy between CeO_2_-GO(4:1)/EP and corrosion inhibitor in simulated seawater solution with different concentrations (20%, 40%, 60%) were analyzed and illustrated by Optical Microscope (OM) characterization, Open Circuit Potential (OCP), Electrochemical alternating current Impedance Spectroscopy (EIS), Mott–Schottky curve and Tafel curve. The results indicated that CeO_2_-GO(4:1) nanocomposites showed good corrosion resistance in a marine environment. This research lays a solid theoretical foundation for the application of cerium oxide-modified graphene oxide anticorrosive coating in marine engineering.

## 1. Introduction

The total ocean area on the earth is about 360 million square kilometers, which is much larger than the land area, accounting for about 71% of the earth’s surface area. The 21st century is a new century for mankind to develop and utilize the ocean. The ocean is rich in resources, and the development of human society will inevitably rely more and more on the ocean [1,2]. However, the marine environment is a complex corrosive environment [3,4]. In this environment, seawater itself is a strong corrosive medium. At the same time, waves and tidal currents produce low-frequency reciprocating stress and impact on metal components, as well as marine microorganisms, attached organisms, and their metabolites, which all directly or indirectly accelerate the corrosion process [5]. The corrosion loss of metal materials in the marine environment is quite serious. Different regions and different depths in the same region often have different seawater concentrations [4,6,7,8]. Different concentrations of seawater contain different proportions of inorganic salts. Oxygen, sea salt particles, heavy metal ions, sulfur ions brought by seawater movement, and some organic substances as ligands will promote the formation of rust spots on the surface of the metal matrix and accelerate the metal corrosion [9,10,11]. The survey shows that the annual loss of ocean corrosion in the United States is 4% of its gross national product and exceeds the total economic loss caused by other natural disasters. As the scale of the marine industry continues to expand, metal marine corrosion will cause more serious economic losses. Thus, corrosion protection of metal materials in the marine environment is particularly important.

For the protection of metal materials in the marine environment, the main method is to cover the surface of the metal with an anti-corrosion coating. Corrosion inhibitor is also widely used because of its good anti-corrosion effect on metal. With the rapid development of marine industry, marine anti-corrosion coatings are also developing in the direction of high performance and environmental protection. The research and promotion of marine anti-corrosion coatings have important practical and economic significance. Gao et al. [12] studied the durability of epoxy-coated reinforced concrete through fatigue loading and saltwater corrosion tests and found that epoxy coating can effectively protect steel bars from corrosion. Huang et al. [13] studied the influence of polyurea coatings on the permeability of chloride ions through dry circulation and natural immersion diffusion methods, and the results showed that polyurea coatings could effectively reduce the permeability and diffusion coefficient of chloride ions in concrete. Pang [14] studied the change of corrosion resistance of Q235B steel plate protected by polyurea coating in accelerated corrosion process with scratch and found the corrosion resistance of Q235B steel plate in seawater solution decreases first and then increases slightly. Tsai et al. [15] added different content of graphene to polyurethane (PU) to prepare a series of polymer composite coatings. The results indicated that adding an appropriate amount of graphene to PU coating could block the penetration of water and oxygen and significantly enhance its anti-corrosion and wear resistance comprehensive performance. Yu et al. [16] prepared functionalized cubic boron nitride (FC-BN) and functionalized hexagonal boron nitride (FH-BN) reinforced epoxy resin coatings and found nanofillers incorporated into polymers can improve the hardness and toughness of the composite coating. Arash et al. [17] doped graphene-based carbon hollow spheres (CHSs) fabrication with 2-mercaptobenzimidazole (MBI) and found an improvement in the adhesion loss of the epoxy coating, ca. 58%, was observed in the presence of 3 wt.% MBI@CHSs.

Graphene oxide (GO) is the oxide of graphene, which has a good barrier effect in the medium environment [18]. Cerium oxide (CeO_2_) can be used as a nano-sheet layered material [19,20]. The study [21] has shown that when the mass ratio of CeO_2_ to GO is 4:1, the anti-corrosion effect of epoxy(EP) coating with CeO_2_-GO nanocomposites is very significant. CeO_2_ is evenly distributed on the GO surface, which improves the agglomeration of GO. Corrosion inhibitors are highly praised for their simple production process [22,23] and low cost [24,25], but their protective effects on metals in complex marine environments are not obvious. 

Our team’s previous research found that the CeO_2_-GO(4:1) nanocomposite coating has excellent anticorrosive performance. In this paper, CeO_2_-GO(4:1) nanocomposites were prepared by the hydrothermal synthesis method, and the three-electrode method was used for electrochemical tests. The corrosion situation was observed by optical microscope (OM), and the state evolution of CeO_2_-GO(4:1)/EP coating immerged in simulated seawater solution with different concentrations (20%, 40%, 60%) was studied by Open Circuit Potential (OCP), Electrochemical alternating current Impedance Spectroscopy (EIS), Mott–Schottky curve and Tafel curve. We compared the anti-corrosion effect of CeO_2_-GO(4:1) nanocomposite coating and corrosion inhibitor and explored the better effect when CeO_2_-GO(4:1) nanocomposite coating and corrosion inhibitor were used together without mutual exclusion. With the prolongation of immersion time in simulated seawater solution, the coating’s corrosion resistance and corrosion tendency were studied. The research results are expected to promote the engineering application of cerium oxide-modified graphene oxide anticorrosive coating in marine environments.

## 2. Materials and Methods

### 2.1. Raw Materials

Graphene oxide (SE2430W) used in this research was a commercial product purchased from Changzhou Sixth Element Materials Technology Co., Ltd., Changzhou, China. The epoxy (WSR6101 E-44) and epoxy AB glue were supplied by Nantong Xingchen Synthetic Material Co., Ltd., Nantong, China. The corrosion inhibitor used was Lan-826 multi-purpose acid pickling corrosion inhibitor [26] by He’nan Xinyang Chemical Co., Ltd., He’nan, China. It was a light yellow to brownish red transparent liquid, with a Ph of 9, a density of 1.030 g/cm^3^, and a corrosion inhibition rate of greater than 99.3%. Cerium hexahydrate nitrate ((CeNO_3_)_3_∙6H_2_O) was an analytical reagent from Shanghai Aladdin Bio-Chem Technology Co., Ltd., Shanghai, China. Anhydrous strontium chloride (SrCl_2_∙6H_2_O), anhydrous magnesium chloride (MgCl_2_∙6H_2_O), calcium chloride (CaCl_2_), sodium fluoride (NaF), potassium chloride (KCl), sodium bicarbonate (NaHCO_3_), bromide Potassium (KBr), boric acid (H_3_BO_3_), sodium chloride (NaCl) and sodium sulfate (Na_2_SO_4_) were supplied by Chengdu Colon Chemicals Co., Ltd., Chengdu, China. In the experiment, Q235 carbon steel with the size of 5 mm in height and 10 mm in diameter was selected to evaluate the anti-corrosion performance of the developed coatings. 

### 2.2. Preparation of Simulated Seawater of Different Concentrations

The marine environment of the actual engineering structure was more complex, and the salt concentration of seawater in different areas was different, thus the simulated seawater solutions with different concentrations were prepared. According to the mass ratio of the solute in the seawater solution and the mass percentage of the solute as the standard, the simulated seawater solution with the mass percentage concentration of 20%, 40%, and 60% was prepared, respectively [8]. Table 1 shows solute mass in simulated seawater at different concentrations. First, SrCl_2_∙6H_2_O, MgCl_2_∙6H_2_O, and CaCl_2_ were proportionally weighed and dissolved in distilled water to 700 mL. Then, NaF, KCl, NaHCO_3_, KBr, and H_3_BO_3_ were weighed out in proportion to dissolve and diluted to 100 mL in distilled water. Next, NaCl and Na_2_SO_4_ were weighed out into distilled water and measured appropriate amount of the above 2 solutions to dilute to 1000 mL and then adjusted the pH to 8.20. A total of 175 mL corrosion inhibitor was added into the solution directly.

### 2.3. Preparation of CeO_2_-GO(4:1)/EP Coationgs

Firstly, a certain amount of dialyzed GO slurry was weighed and dissolved in the water, and after the stripping process of an ultrasonic cell crushing machine, the thin-flake GO could be obtained. Then, an appropriate amount of cerium hexahydrate nitrate was added to the GO solution and ultrasonically stirred for 30 min, followed by another 30 min of magnetically stirring process, with the ammonia hydroxide added during the stirring process, for better dispersion. After that, the obtained solution was put in a high-pressure reaction kettle with 200 mL polytetrafluoroethylene liner mixed and reacted for 24 h at 180 °C. Finally, the solid phase within the solution was extracted by washing and filtering with deionized water and anhydrous ethanol, and after a drying and ground process, the CeO_2_-GO nanocomposite was produced. 

**Figure 1:**
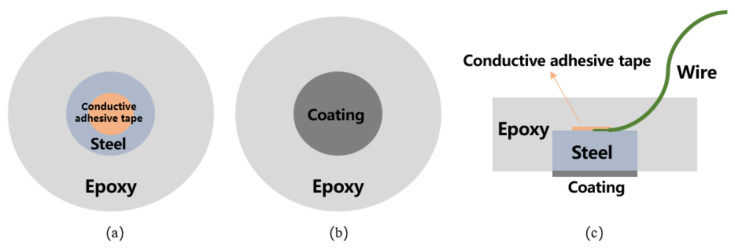
A schematic map of the prepared sample: (**a**) top view; (**b**) bottom view; (**c**) side view.

A cylindrical Q235 carbon steel was applied to examine the anti-corrosion performance of the developed CeO_2_-GO coating. The sample was prepared with the following procedures. Firstly, a copper conductor was affixed to the one side surface of the Q235 carbon steel, and the steel was centered into a cylindrical plastic mold (approximately 20 mm in diameter, 15 mm in height) with another side (no copper conductor attached) facing down. Then the AB epoxy was poured into the mold until it was roughly over the top surface of the steel sample and the mixture was kept in the mold for 24 h until the epoxy hardened. Then, the sample was demolded, and the bottom surface of the sample was ground and polished with abrasive papers. The sample was ultrasonically cleaned in alcohol to remove the debris caused by the grinding and polishing procedure. CeO_2_-GO(4:1) nanocomposites were prepared by a hydrothermal synthesis method [21]. Then, 4 g epoxy was measured, and a certain amount of CeO_2_-GO(4:1) (0.5 wt% of the epoxy mass) was added to the epoxy after a 30 min ultrasonic dispersion process. After that, the mixture was magnetically stirred for 30 min for better mixing, and the acetone was removed by evaporation. 1 g curing agent was added to the above mixture, and the mixture was stirred before it was stored in a vacuum drying oven to drive out the bubbles. Finally, the mixture was coated on the bottom surface of the Q235 carbon steel with a coating machine, and the coating thickness was controlled at 100 μm by a coating thickness gauge. The CeO_2_-GO(4:1)/EP coating was obtained after being cured at room temperature for 24 h. A schematic map of how the sample was prepared is shown in Figure 1. The samples were placed in the simulated seawater solution to investigate the effect of the coating.

For comparison, 4 samples denoted as G1, G2, G3, and G4, were prepared, with G1 being EP coating, G2 being the EP coating with corrosion inhibitor added, G3 being CeO_2_-GO(4:1)/EP coating, and G4 being the CeO_2_-GO(4:1)/EP coating with corrosion inhibitor added. The performance of G1, G2, G3, and G4 was compared to prove the excellent corrosion resistance of CeO_2_-GO(4:1)/EP coating in different concentrations of simulated seawater solution. Table 2 shows the coating conditions of G1, G2, G3, and G4.

### 2.4. Testing Procedures

#### 2.4.1. Micromorphological Characterization

**Figure 2:**
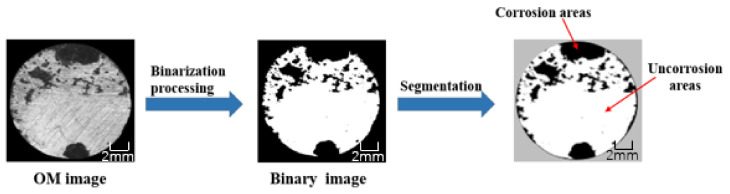
The main process of analyzing the OP image.

The optical microscope uses optical principles to magnify and image tiny objects to observe the corrosion area on the surface of the steel bar. The sample was uncovered the surface coating of the immersed steel bar and wiped the surface solution with filter paper. After drying, the steel bar was placed on the workbench, and visible light beams were used to locally scatter or reflect on the surface of the steel bar, showing corroded and uncorroded areas. Figure 2 shows the main process of analyzing the OP image. After image processing, the corrosion area can be obtained, and the proportion of corrosion can be calculated according to Equation (1), which can be used as an index to quantitatively characterize the effect of corrosion prevention.

(1) $$\gamma =\frac{S_{corr}}{S}$$

where represents the corrosive area, *S* represents the area of the steel bar exposed to the coating. 

#### 2.4.2. Electrochemical Testing

The electrochemical workstation of CHI-760E type was used for electrochemical testing, with the saturated calomel electrode being the reference electrode and the platinum electrode as the auxiliary electrode. The working electrode was Q235 carbon steel coated with epoxy resin. The scanning range of open circuit potential (OCP) was −200–1200 mV. The frequency range of EIS test was 10^5^–10^−2^ Hz, and the amplitude was 10 mV. The scanning range of the potentiometric polarization curve was −300–300 mV, and the scanning rate was 1 m V/s. The adopted Mott–Schottky frequency was 1000 Hz, and the scanning interval was −1–0.5 V. The samples of G1, G2, G3, and G4 were tested. Through the change of anti-corrosion performance of the coating and corrosion inhibitor in different soaking periods, the trend of the anti-corrosion efficiency of the coating can be judged.

## 3. Anti-Corrosion Performance

### 3.1. Open Circuit Potential(OCP) Analysis

**Figure 3:**
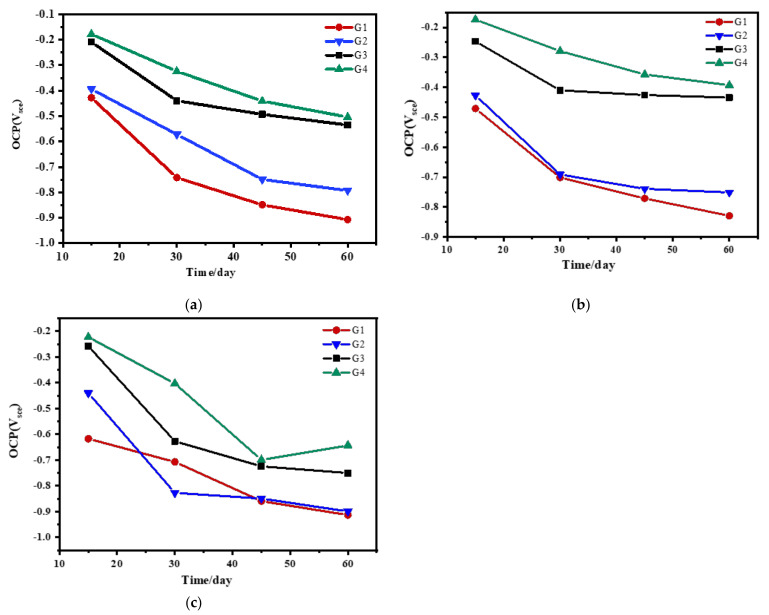
Each coating’s OCP for immersion for 60 days: (**a**) 20% Seawater; (**b**) 40% Seawater; (**c**) 60%.

The OCP results of four coatings eroded in the simulated seawater solution of different concentrations for 60 days are shown and compared in Figure 3. When immersed for 15 days, the open circuit potential of the coating doped with CeO_2_-GO(4:1) was measured to be small, and the difference was not significant, indicating that the seawater hardly penetrated into the coating to contact with the metal substrate at this time. However, the open circuit potential of the coating without CeO_2_-GO(4:1) was close to −400 mV, and the anti-corrosion performance of the coating without nano-filler was significantly reduced. Although the corrosion inhibitor was added, the effect was not obvious. With the extension of immersion time and the increase of salinity in the solution, the open circuit potentials of G1, G2, G3, and G4 decreased significantly. At this time, the seawater completely penetrated through the coating and reached the metal surface. Moreover, the compactness of each coating, namely the shielding ability to electrolyte and corrosive particles, decreased a lot. In general, the open circuit potential of G3 decreased but was significantly higher than G2, which indicated that nanocomposites were involved in metal corrosion. The incorporation of corrosion inhibitors also slow down galvanic corrosion but have poor protection for metals. The open-circuit potential of G4 is slightly higher than that of G3, which indicates that the synergistic effect between CeO_2_-GO(4:1) nanocomposite and corrosion inhibitor improves the corrosion resistance.

### 3.2. Electrochemical Alternating Current Impedance Spectroscopy (EIS) Analysis

**Figure 4:**
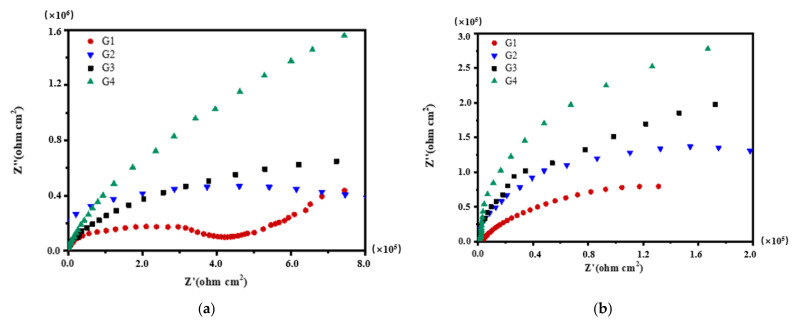

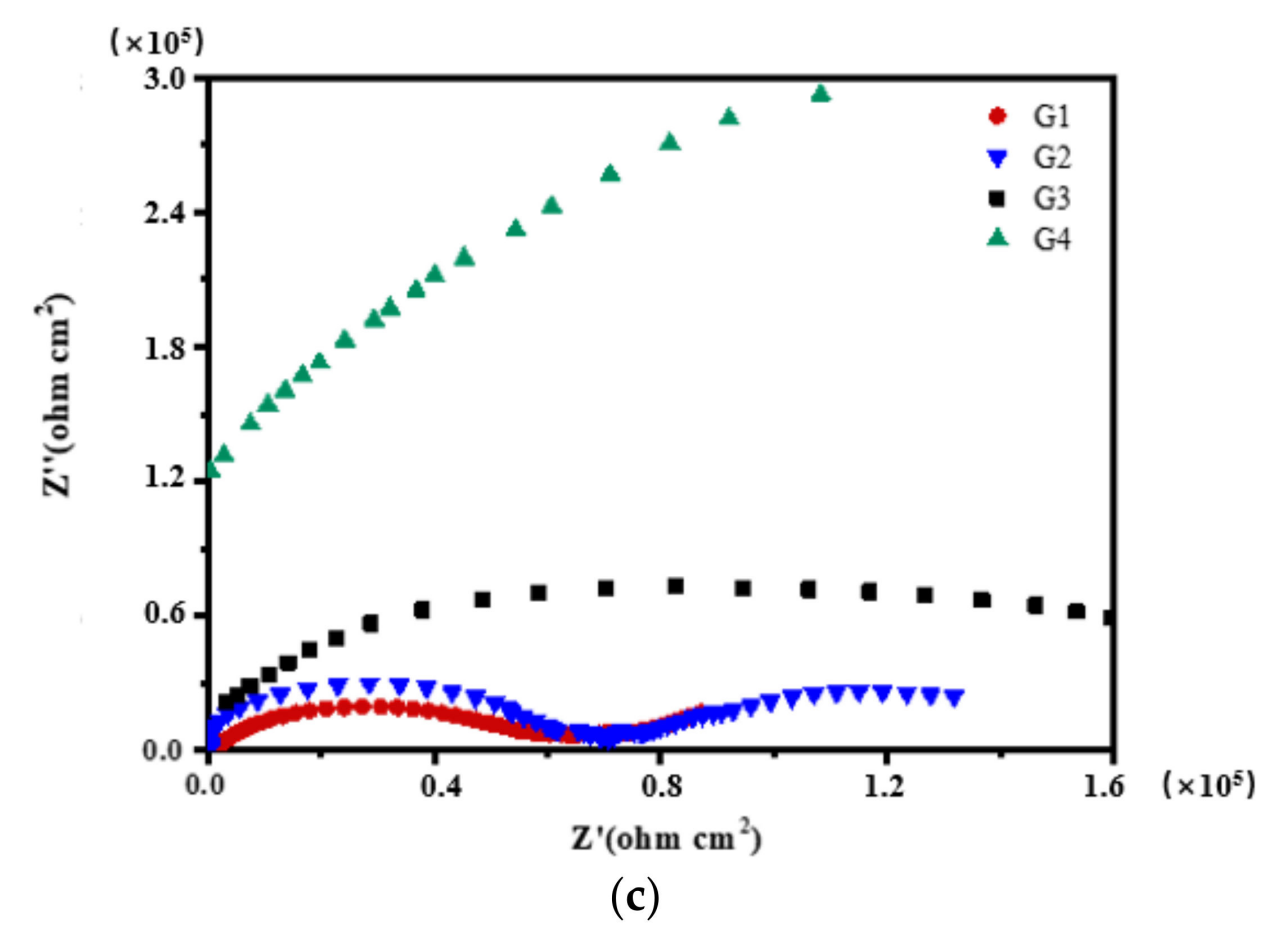
Each coating’s Nyquist pattern for immersion for 15 days: (**a**) 20% Seawater; (**b**) 40% Seawater; (**c**) 60% Seawater.

**Figure 5:**
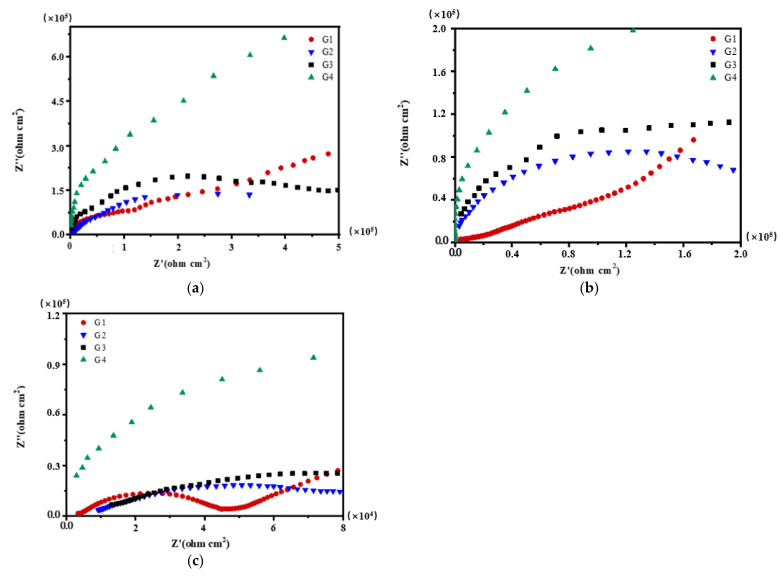
Each coating’s Nyquist pattern for immersion for 30 days: (**a**) 20% Seawater; (**b**) 40% Seawater; (**c**) 60% Seawater.

**Figure 6:**
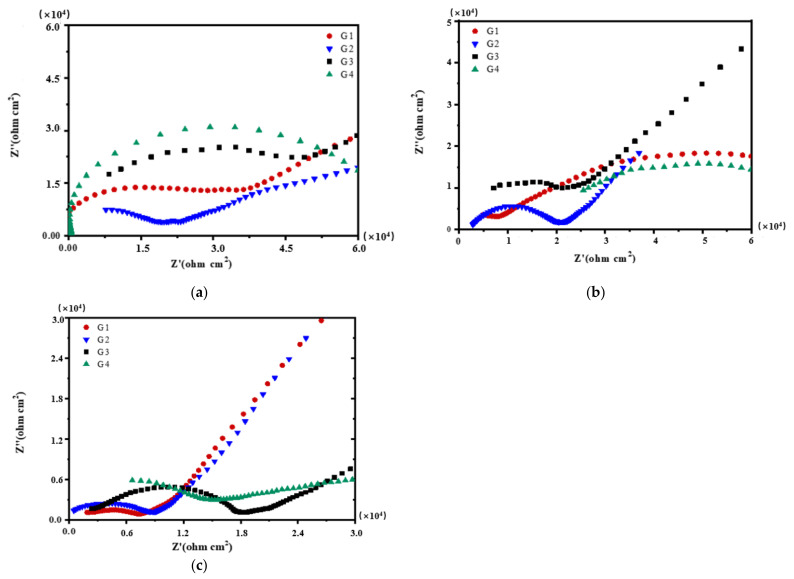
Each coating’s Nyquist pattern for immersion for 45 days: (**a**) 20% Seawater; (**b**) 40% Seawater; (**c**) 60% Seawater.

**Figure 7:**
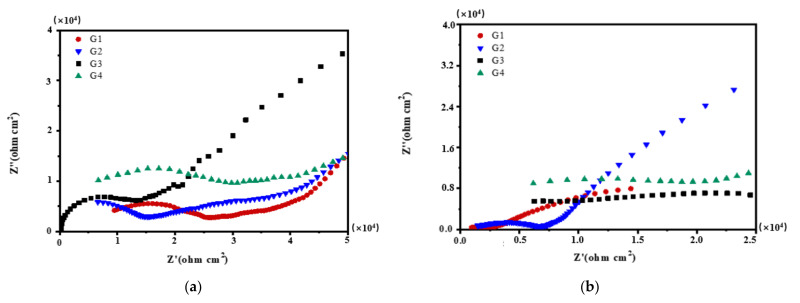

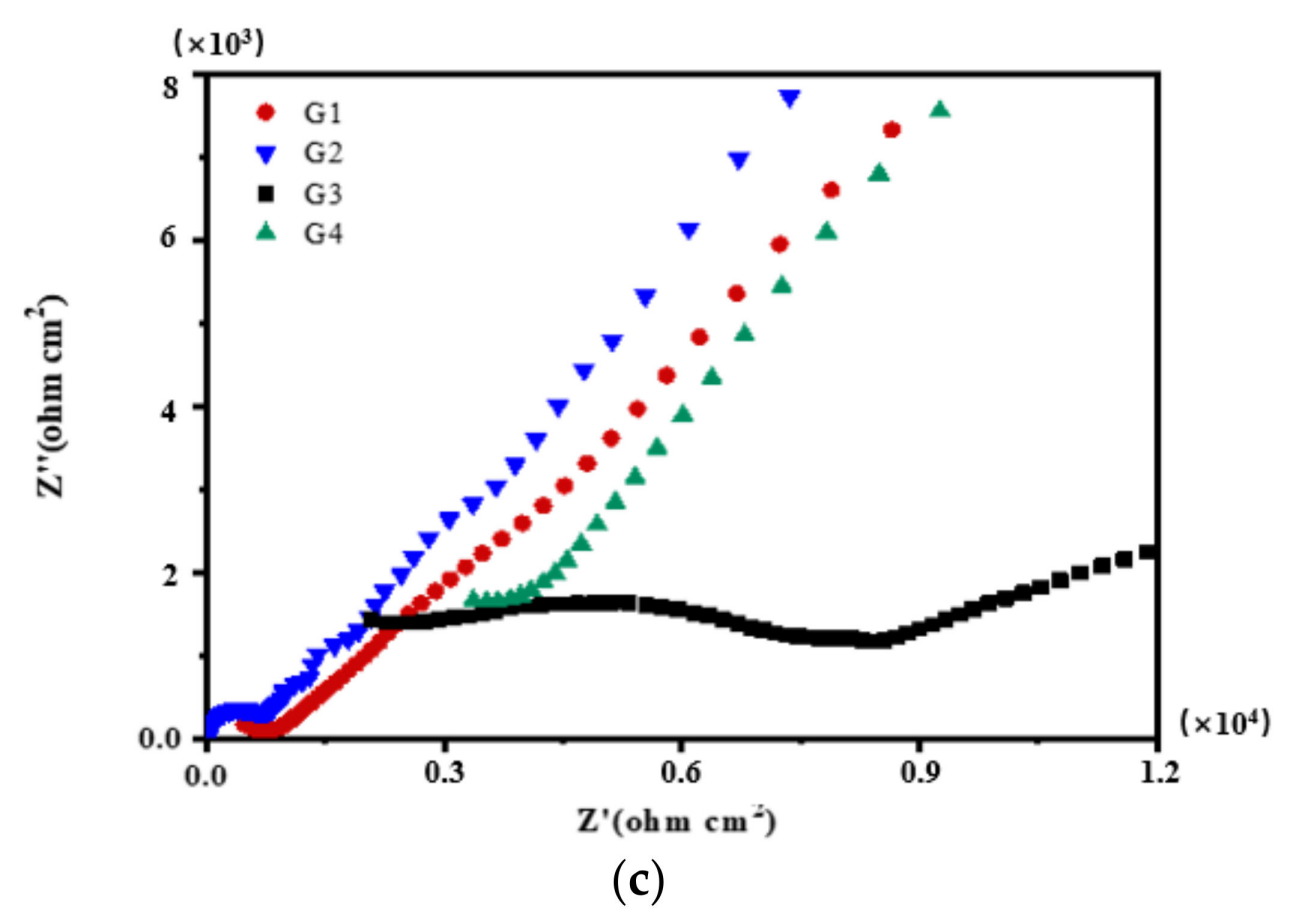
Each coating’s Nyquist pattern for immersion for 60 days: (**a**) 20% Seawater; (**b**) 40% Seawater; (**c**) 60% Seawater.

Figure 4, Figure 5, Figure 6 and Figure 7 show Nyquist patterns of carbon steel coated with different coatings immersed in the simulated seawater solutions of different concentrations for 60 days. Figure 4 shows Nyquist patterns of G1, G2, G3, and G4 immersed in simulated seawater solutions of different concentrations for 15 days. The four coatings all present a semicircle shape of impedance in the low-frequency region, and the radius of the capacitor reactance arc of the coatings is very large at this time. The protection mainly depends on the shielding of epoxy. Figure 5 shows Nyquist patterns of G1, G2, G3, and G4 immersed in simulated seawater solutions of different concentrations for 30 days. At this time, the radius of the capacitive reactance arc decreased gradually. The greater the concentration of seawater is, the smaller the impedance value of the coating is, and the corrosion intensifies. Figure 6 shows Nyquist patterns of G1, G2, G3, and G4 immersed in simulated seawater solutions of different concentrations for 45 days. With the prolongation of immersion time, more Cl^−^, SO_4_^2−^ and dissolved oxygen in the solution enter and diffuse from the micropores on the surface of the coating and the capillary channels inside the coating. The rapid electron transfer between the corrosive medium, coating, and metal substrate intensifies the corrosion, and the radius of the capacitive reactance arc of the curve decreases in a regular manner. Refer to the existing research [12,15], impedance spectroscopy can feedback the information of the passivation film on the metal surface. In Figure 6a, the impedance value of G1 is greater than that of G2. The reason might be that with the aggravation of corrosion, a layer of product film formed by corrosion products accumulated on the metal surface foams and damages. Figure 7 shows Nyquist patterns of G1, G2, G3, and G4 immersed in simulated seawater solutions of different concentrations for 60 days. In the later stage of immersion, the capacitive arc radiuses of the four coatings decrease sharply, and some coatings disappear the Warburg tail in the low-frequency region, and two arcs appear. At this time, the protective performances of the coatings basically disappear. 

In the same concentration of seawater, with the prolonging of immersion time, the radius of capacitive reactance arc and the value of coating impedance decrease, and the corrosion resistance of the coating becomes worse and worse. With the increase of salinity in the solution for the same soaking time, the coating impedance value decreases, and the corrosion resistance of the coating becomes worse and worse. Both are due to corrosive particles and electrolyte infiltration in the solution. In either case, the corrosion resistance of the nanocomposite doped with CeO_2_-GO(4:1) is better than that of the corrosion inhibitor. The reason may be that the modified CeO_2_ itself has anti-corrosion performance, thus the modified composite material not only solves the problem of GO stacking and agglomeration well but also increases the physical shielding ability of two-dimensional graphene. In addition, CeO_2_ hydrolyzes with H_2_O during the immersion process, resulting in cerium hydroxide (Ce(OH)_4_), which is insoluble in water and collects on the surface of the coating micropores and capillary channels inside the coating, slowing down the penetration of H_2_O, Cl^−^ and SO_4_^2−^, indicating that CeO_2_-GO affects the corrosion of the metal substrate to a certain extent.

**Figure 8:**
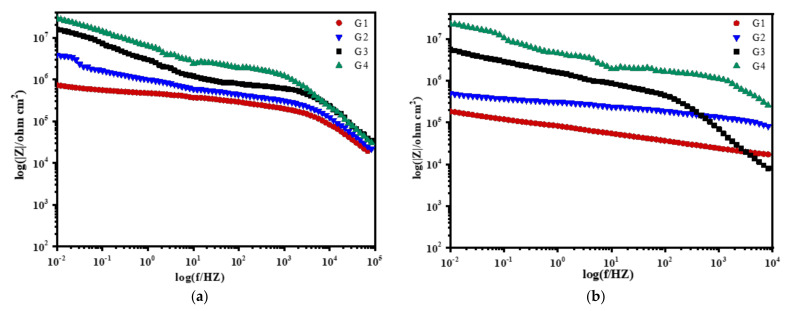

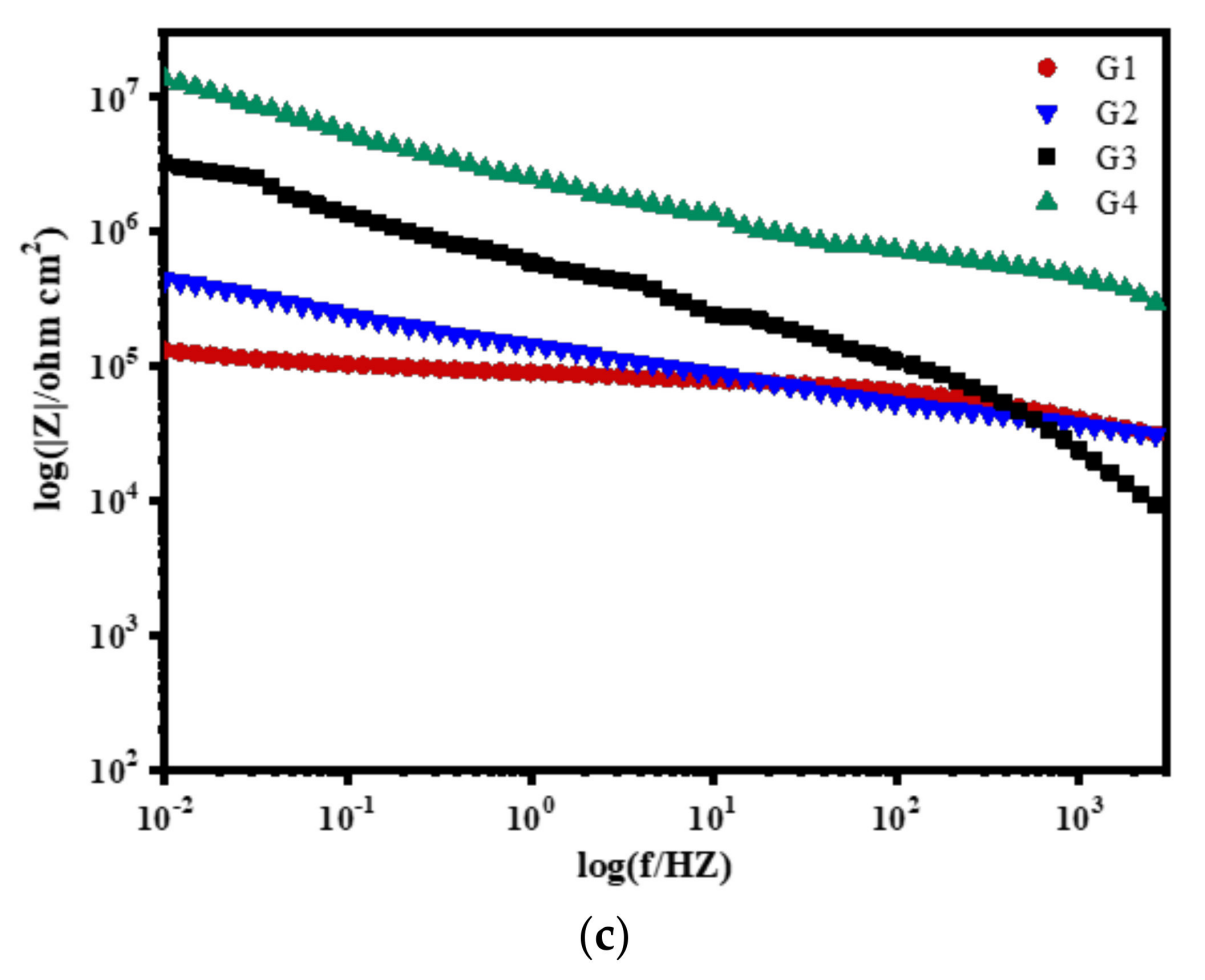
Each coating’s Bode pattern for immersion for 15 days: (**a**) 20% Seawater; (**b**) 40% Seawater; (**c**) 60% Seawater.

**Figure 9:**
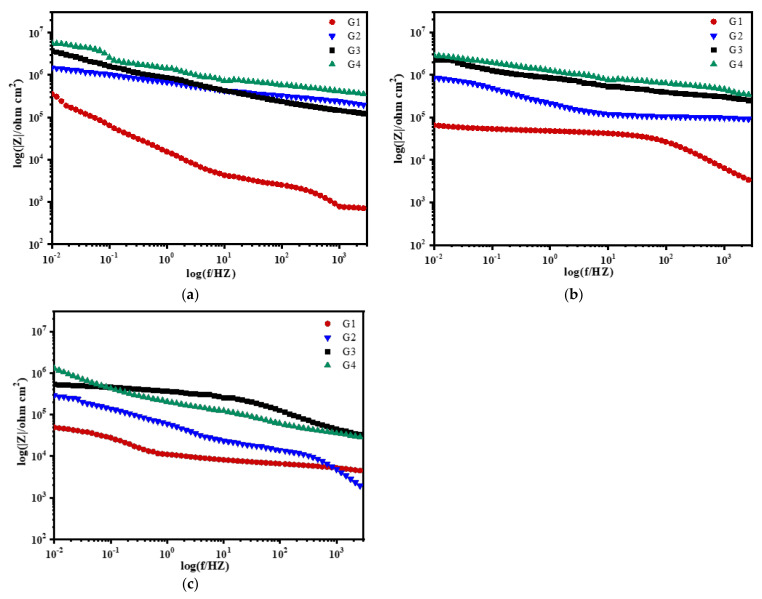
Each coating’s Bode pattern for immersion for 30 days: (**a**) 20% Seawater; (**b**) 40% Seawater; (**c**) 60% Seawater.

**Figure 10:**
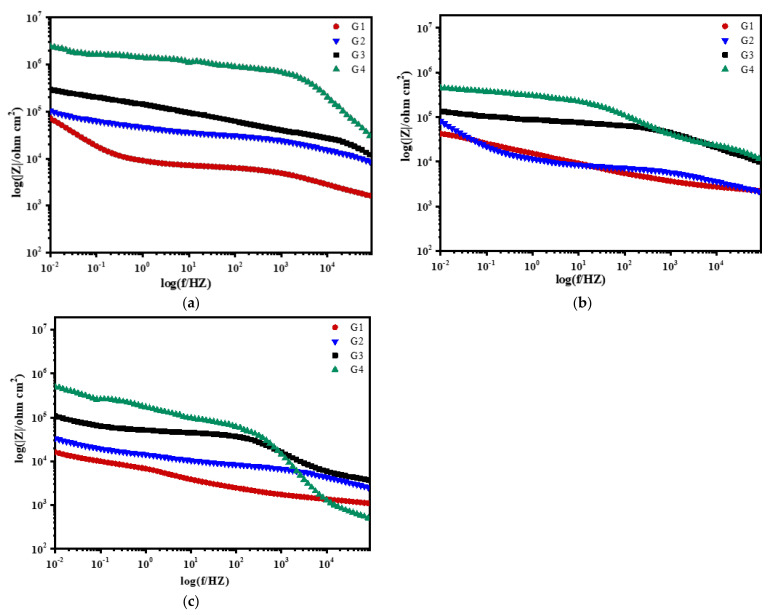
Each coating’s Bode pattern for immersion for 45 days: (**a**) 20% Seawater; (**b**) 40% Seawater; (**c**) 60% Seawater.

**Figure 11:**
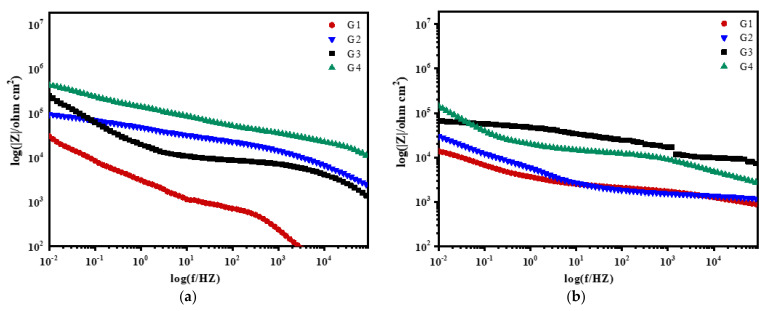

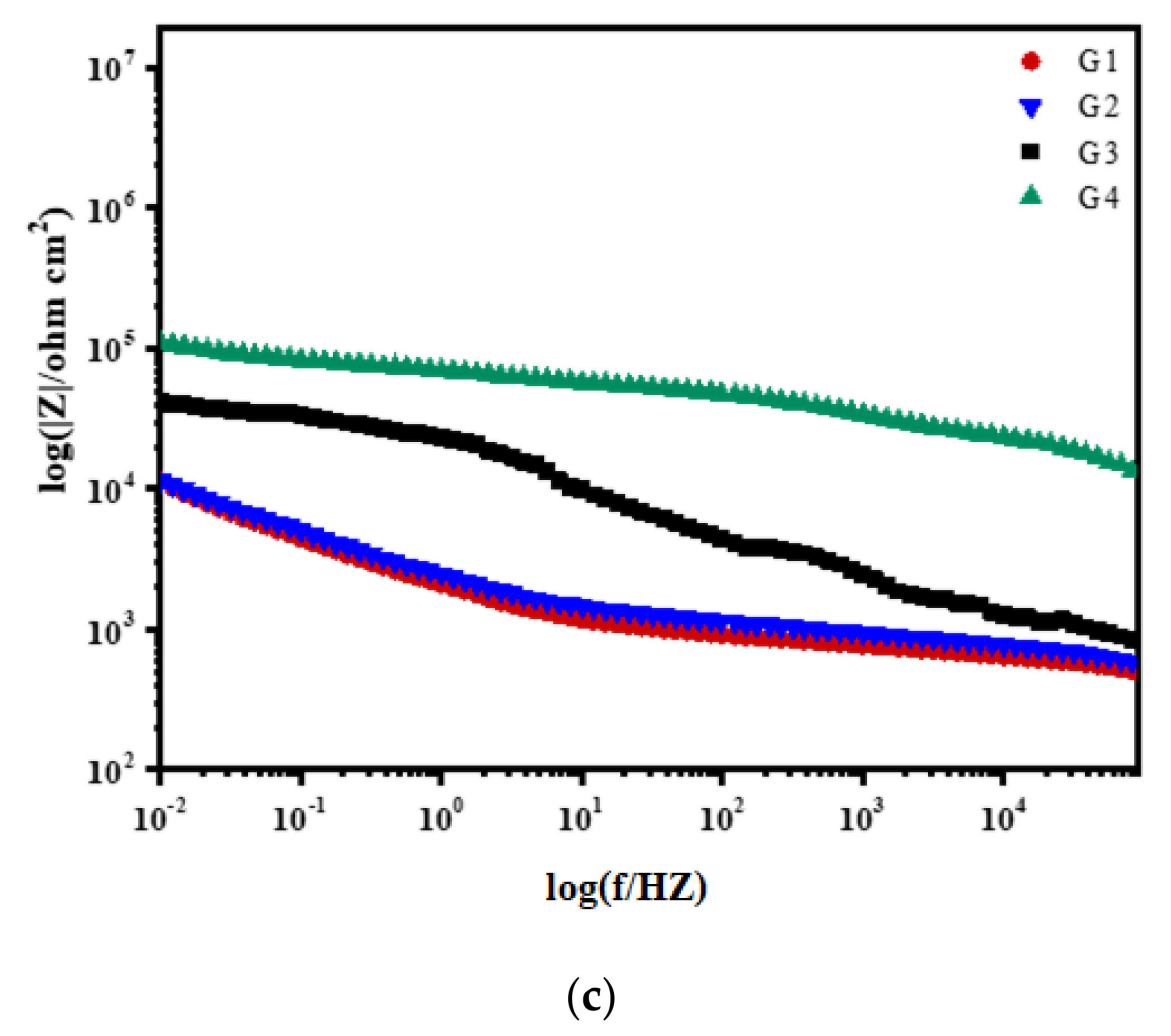
Each coating’s Bode pattern for immersion for 60 days: (**a**) 20% Seawater; (**b**) 40% Seawater; (**c**) 60% Seawater.

Figure 8, Figure 9, Figure 10 and Figure 11 show Bode patterns of carbon steel coated with different coatings immersed in the simulated seawater solutions of different concentrations for 60 days. The resistance value of the low-frequency region in the Bode figure is the impedance modulus value of the coating. The anti-corrosion performance of the four coatings can be compared by observing the resistance value of the low-frequency region. The higher the modulus value of low-frequency area is, the better the protective performance of the coating is [27]. As the soaking time extension and the increase of solution concentration, G2 log|Z|0.01 HZ value from 3.89 × 10^9^ Ω/cm^2^ (15 d, 20% seawater) down to 1.14 × 10^4^ Ω/cm^2^ (60 d, 60% seawater), decreases significantly. The modulus decline of G3 is smaller than that of G2, which indicates that the corrosion resistance of G3 is less lost than that of G2. It could also be analyzed from the results that G4 can maintain good corrosion resistance in a high concentration corrosive environment, showing well corrosion resistance. With the increase of the concentration of simulated seawater, the salt content in the solution increases, and the impedance values of the four coatings decrease obviously. As the extension of soaking time, the corrosion resistance of the four coatings decreases significantly, and the various trends of the coatings are consistent with the analysis results of Nyquist patterns.

### 3.3. Mott–Schottky Curve Analysis

**Figure 12:**
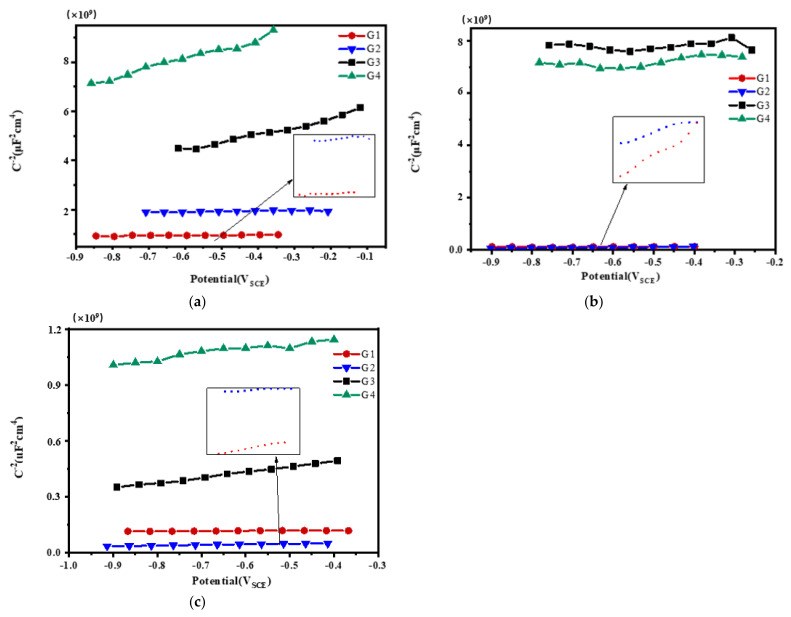
Each coating’s Mott–Schottky pattern for immersion for 15 days: (**a**) 20% Seawater; (**b**) 40% Seawater; (**c**) 60% Seawater.

**Figure 13:**
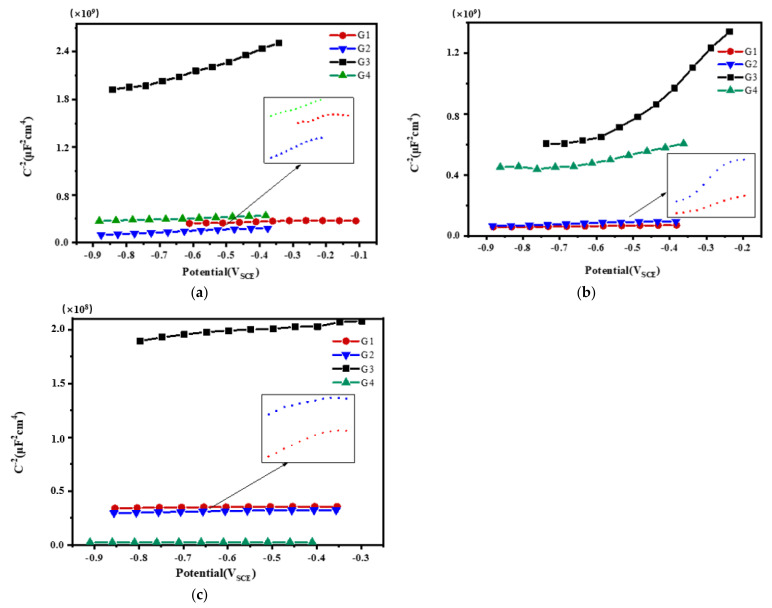
Each coating’s Mott–Schottky pattern for immersion for 30 days: (**a**) 20% Seawater; (**b**) 40% Seawater; (**c**) 60% Seawater.

**Figure 14:**
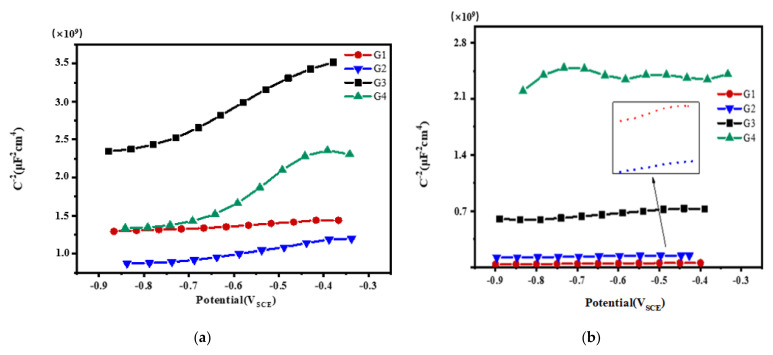

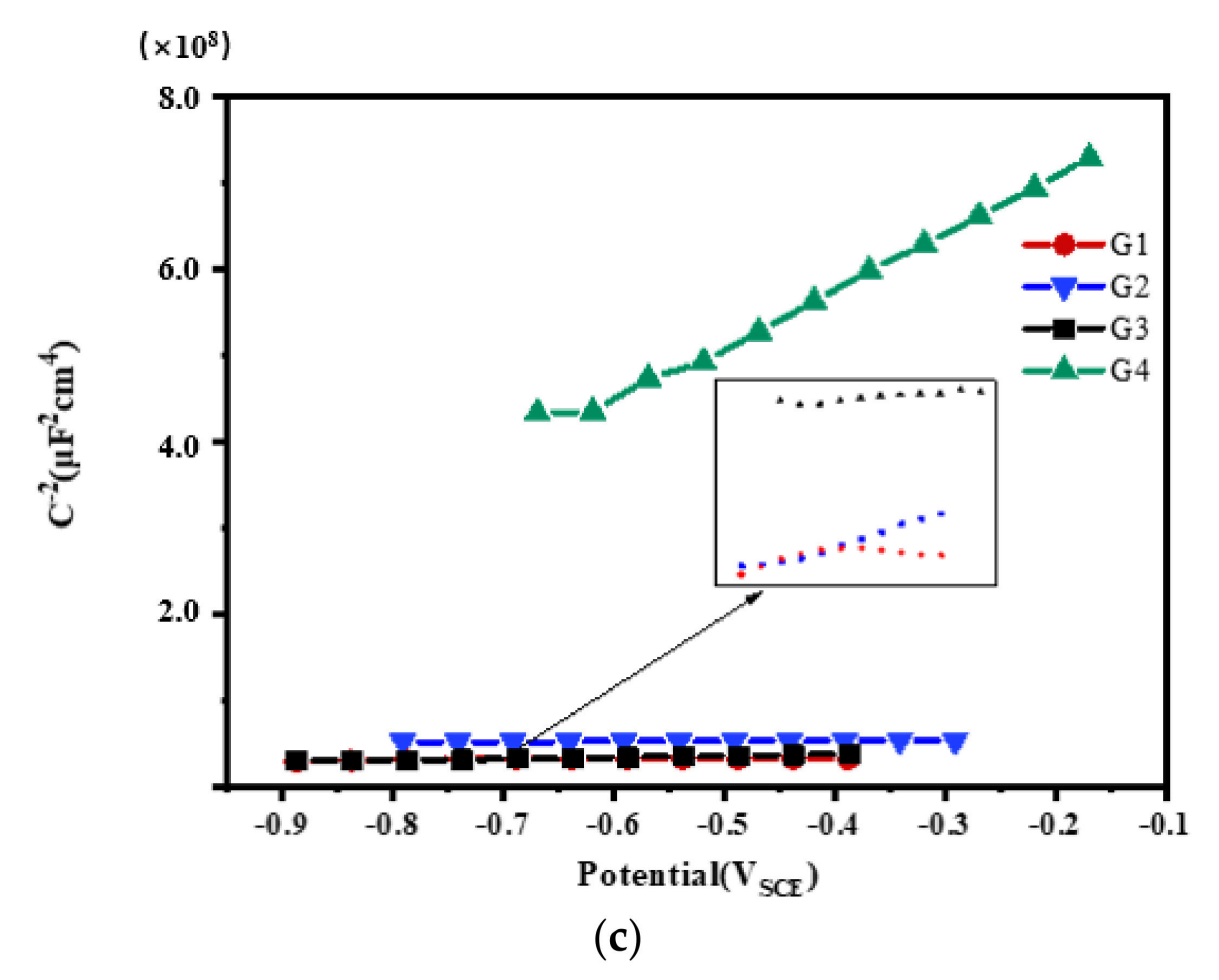
Each coating’s Mott–Schottky pattern for immersion for 45 days: (**a**) 20% Seawater; (**b**) 40% Seawater; (**c**) 60% Seawater.

**Figure 15:**
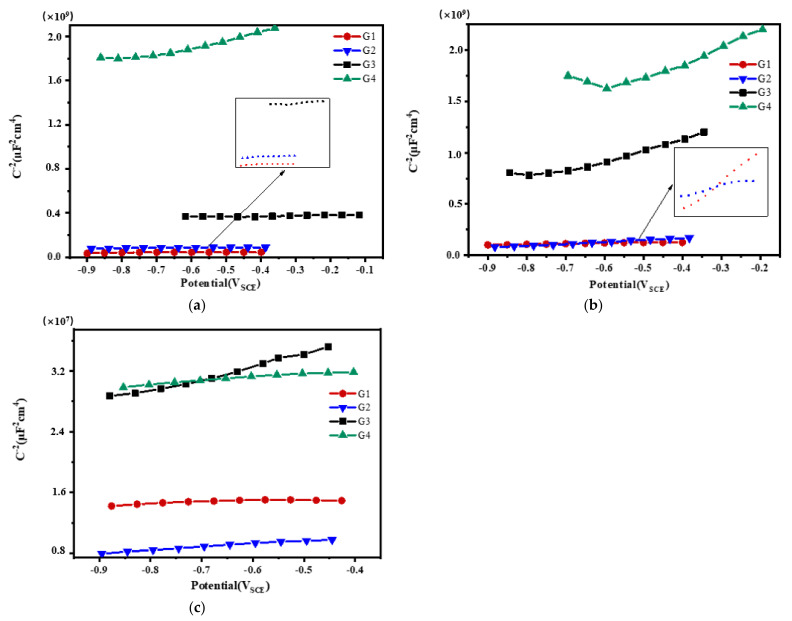
Each coating’s Mott–Schottky pattern for immersion for 60 days: (**a**) 20% Seawater; (**b**) 40% Seawater; (**c**) 60% Seawater.

Mott–Schottky reflects the comprehensive properties of the electrode surface. The different movement forms of deeply charged particles inside the coating make the electrochemical properties and corrosion behavior of the electrode different [28]. Figure 12, Figure 13, Figure 14 and Figure 15 show Mott–Schottky patterns of carbon steel coated with different coatings immersed in the simulated seawater solutions of different concentrations for 15 days, 30 days, 45 days and 60 days. Table 3, Table 4 and Table 5 show the carrier densities of G1, G2, G3 and G4 immersed in simulated seawater solutions of different concentrations. It can be seen that the slope of the Mott–Schottky curves and the carrier densities of the four coatings are positive at each stage of immersion in different solutions, which are characterized by n-type semiconductors. The movements of corrosive particles do not significantly change the donor density and receiver density of the protective film. As can be seen from Figure 12 and Table 3, Table 4 and Table 5, with the increase of salinity in the solution, the slope of Mott–Schottky curves of the four coatings increase significantly, and the carrier densities increase. The corrosion rates of reinforcement also increase. However, in Figure 12a, the capacitance of the space charge layer of the coating fluctuates, indicating that the thickness of the space charge layer changes due to the change of carrier density inside the coating. In addition, the slope of Mott–Schottky curve of G3 and G4 doped with CeO_2_-GO nanocomposite is significantly higher than that of the coating doped with corrosion inhibitor, and the carrier density is relatively small, indicating that CeO_2_-GO nanocomposite reduces the carrier movement rate inside the coating and plays a good isolation effect on the external water and ions. As shown in Figure 13, the Mott–Schottky curves’ slope decrease as the solution concentration increases with the extension of immersion time, and the changes of the coatings mixed with corrosion inhibitor are obvious. This indicates that with the infiltration of electrolyte and corrosive particles, the composite coating doped with CeO_2_-GO has a good anti-corrosion effect and the coating mixed with corrosion inhibitor has a poor anti-corrosion effect. It can be seen from Figure 14 and Table 3, Table 4 and Table 5 that the four coatings continue to corrode in the middle and late stages of immersion, but the accumulation of corrosion product on the surface of the metal substrate blocks the channel for ion diffusion to the electrode surface, reducing the probability of metal being oxidized. The corrosion rates of the four coatings decrease significantly, and the slopes of the Mott–Schottky curves decrease, and the carrier densities increase but to a lesser extent. It can be seen from Figure 15 that in the later stage of the immersion, as more Cl^−^, SO_4_^2−^ and dissolved oxygen in the seawater erode the coating, the carrier migration speed inside the coating is extremely high, and the thickness of the space charge layer is extremely thin. The coating basically loses its protective ability. However, during the immersion process, the water solubility of CeO_2_ reduces the conductivity of the coating to electrons and greatly reduces the tendency of the base metal to lose electrons. It can be seen that the nano-CeO_2_ modified GO greatly improves the anti-corrosion ability of the epoxy coating.

### 3.4. Tafel Curve Analysis

**Figure 16:**
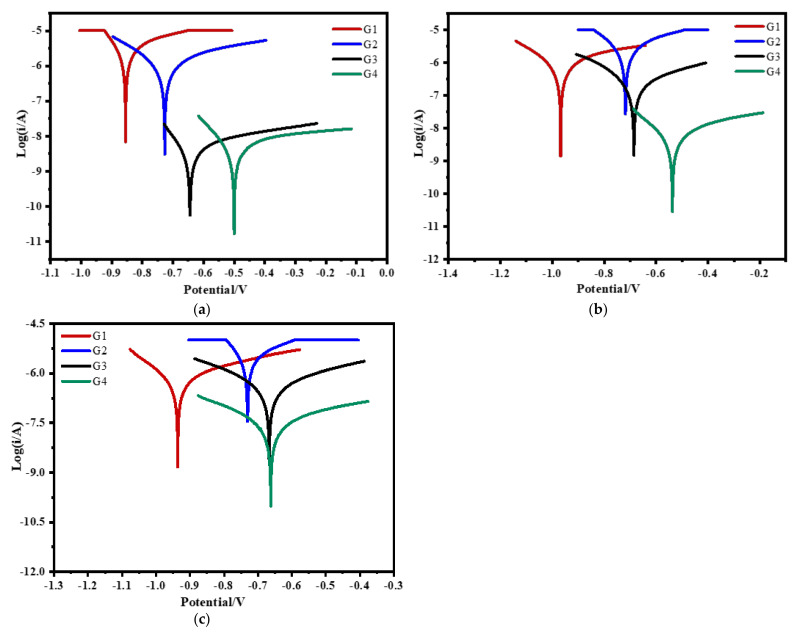
Each EP/CeO_2_-GO coating’s Tafel curve for 60 days: (**a**) 20% Seawater; (**b**) 40% Seawater; (**c**) 60% Seawater.

Figure 16 shows the Tafel curve of carbon steel coated with different coatings immersed in the simulated seawater solutions of different concentrations for 60 days. It can be seen from the testing results that the salinity in seawater has an obvious influence on the coating. The higher the solution concentration is, the greater the self-corrosion current density of the coating is, and the worse the shielding ability of the coating to corrosion particles and dissolved oxygen is. Table 6 shows corrosion potential E_corr_, corrosion current density I_corr_, polarization resistance R_p_ and coating protection efficiency η of G1, G2, G3 and G4 in seawater of 20% concentration for 90 days. The corrosion density of G4 is the smallest, which is only 1.543 × 10^−9^ A/cm^2^, and the coating protection rate is 99.93%. Compared with G3, the polarization resistance of G4 is larger, but the gap is smaller. It can be seen that corrosion inhibitors are involved in metal protection, but the effect is not obvious. However, compared with G2, the corrosion current density of G3 is lower than two or three orders of magnitude, indicating that CeO_2_-Go nanocomposite material not only makes the coating denser but also reduces the micropores in the coating and has a good anti-corrosion performance. The differences of G1, G2, G3, and G4 in Table 7 and Table 8 are the same as those in Table 6, which well proves that the anti-corrosion effect of CeO_2_-GO nanocomposite is better than that of corrosion inhibitor.

## 4. Corrosion Morphology Comparison

**Figure 17:**
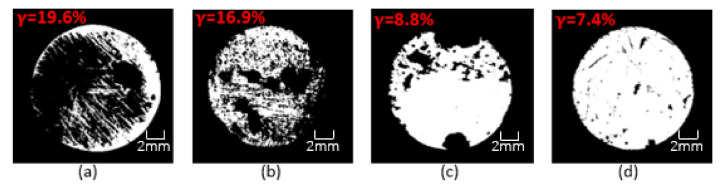
Binary image of each coating in seawater of 20% concentration for 60 days: (**a**) G1; (**b**) G2; (**c**) G3; (**d**) G4.

**Figure 18:**
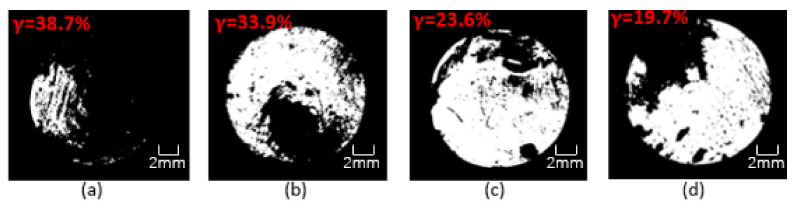
Binary image of each coating in seawater of 40% concentration for 60 days: (**a**) G1; (**b**) G2; (**c**) G3; (**d**) G4.

**Figure 19:**
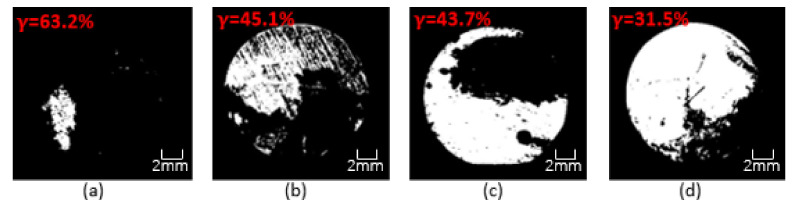
Binary image of each coating in seawater of 60% concentration for 60 days: (**a**) G1; (**b**) G2; (**c**) G3; (**d**) G4.

This research used OM to clearly observe the corrosion on the surface of Q235 steel bars and then evaluated the anti-corrosion performance of the coating. Figure 17, Figure 18 and Figure 19 show the binary image of each coating in seawater of different concentration for 60 days, and the corrosion rate is marked. It could be clearly seen from the figures that the coating had been destroyed and lost its protective ability after 60 days of soaking. The black shaded area of the steel bar surface is the corroded surface, and the white area is the uncorroded part. The corroded surface is concentrated and extended from the edge to the center, and part of the corrosion is concentrated on the polished surface of the steel bar. As the immersion time increases, the protective ability of the coating gradually decreases. As the concentration of the simulated seawater solution increases, the corrosion situation becomes more and more serious. OM image analysis shows that the CeO_2_-GO(4:1) nanocomposite and the addition of corrosion inhibitor could improve the anti-corrosion ability of epoxy coating and the anti-corrosion effect of CeO_2_-GO(4:1) nanocomposite is better. CeO_2_-GO(4:1) nanocomposite with corrosion inhibitor also shows excellent corrosion resistance.

**Figure 20:**
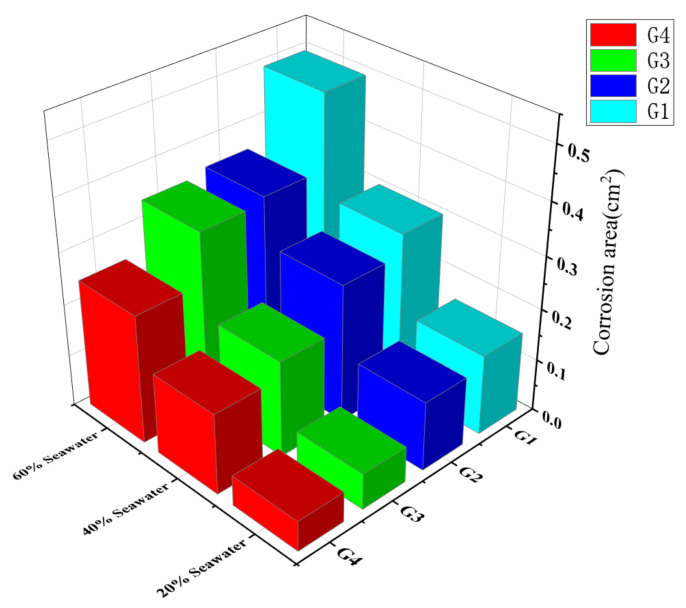
The corrosion area of G1, G2, G3, and G4 immersed in simulated seawater solutions.

In order to quantitatively analyze the corrosion of the steel bar surface, the corrosion area of Q235 carbon steel is calculated [26]. Figure 20 shows the corrosion area of G1, G2, G3, and G4 immersed in three simulated seawater solutions of different concentrations for 60 days.

It can be seen from the calculation results in Figure 20 that when the concentration of the simulated seawater solution is 20%, the corrosion area of G1 is 0.154 cm^2^, and the corrosion area of G2 is 0.133 cm^2^, and there is little difference between the two. However, the corrosion area of G3 is only 0.069 cm^2^, which is about half of the corrosion area of G2 and has a small difference from that of G4. It can be seen that the corrosion resistance of CeO_2_-GO(4:1)/EP coating is obviously better than that of epoxy coating in the environment with corrosion inhibitor added. When the concentration of simulated seawater solution is 40%, the corrosion area of G2 is 0.266 cm^2^, and the corrosion area of G3 is 0.185 cm^2^. The corrosion area of G4 is 0.155cm^2^, indicating that the corrosion damage degree of corrosion inhibitor is higher than that of CeO_2_-GO(4:1)/EP coating. At the same time, CeO_2_-GO(4:1)/EP coating shows more excellent corrosion resistance in simulated seawater solution with corrosion inhibitor added. With the concentration of simulated seawater solution increases gradually, when the concentration of simulated seawater solution is 60%, the advantage of G3 over G2 in corrosion resistance decreases gradually. It can be seen that the protective ability of the coating and corrosion inhibitor is damaged due to the high concentration of simulated seawater solution. The calculation results of the corrosion area are consistent with the analysis results of OM images.

## 5. Conclusions

In this paper, the state evolutions of CeO_2_-GO(4:1)/EP coating during servicing in simulated seawater solution with different concentrations (20%, 40%, 60%) were studied. EP coating, EP coating + corrosion inhibitor, and CeO_2_-GO(4:1)/EP coating + corrosion inhibitor were prepared for comparative study. The following conclusions can be reached based on experimental results.

(1) The addition of CeO_2_-GO(4:1) nanocomposite and corrosion inhibitor can improve the anti-corrosion ability of epoxy coating and the anti-corrosion ability of CeO_2_/GO(4:1) nanocomposite was better. By comparing G3 with G4, we can see that G4 had a slight increase in anti-corrosion efficiency, which indicated that CeO_2_-GO(4:1)/EP coating and corrosion inhibitor were not mutually exclusive and could play a synergistic effect.

(2) The reinforcement corrosion was investigated through OCP, EIS, Mott–Schottky curve, and Tafel curve for 60 days. The results indicated that with the increase of the concentration of simulated seawater solution and the prolongation of immersion time, the corrosion of steel reinforcement intensified. Tafel curve showed that the protective efficiency of coating with CeO_2_-GO(4:1) nanocomposite was much higher than that of pure epoxy coating and corrosion inhibitor, and the protective efficiency was up to 98.87% and 99.93% in 20% seawater. At the same time, CeO_2_-GO(4:1) nanocomposite and corrosion inhibitor can be used together to play a better anti-corrosion effect

(3) Corrosion inhibitor was involved in reinforcement protection, while CeO_2_-GO(4:1) nanocomposite performed better with EP, and the corrosion product film generated by hydrolysis could fill the micropores on the surface of the coating, as well as the capillary channels inside the coating, improving the density of the coating and the anti-corrosion ability of the coating. This study was beneficial to promote the application of cerium oxide–graphene oxide modified anticorrosive coating in ocean engineering and improve the corrosion status of metal. 

## Figures and Tables

**Table 1:** Solute mass in simulated seawater at different concentrations.

Soulte Quality/g	20% Seawater	40% Seawater	60% Seawater
SrCl_2_∙6H_2_O	0.43	1.14	2.58
MgCl∙6H_2_O	112.8	300.81	676.83
CaCl_2_	11.75	31.37	70.59
NaF	0.05	0.14	0.31
KCl	11.84	31.5	71.06
NaHCO_3_	3.43	9.14	20.56
KBr	1.73	4.57	10.29
H_3_BO_3_	0.46	1.23	2.78
NaCL	24.54	24.54	24.54
Na_2_SO_4_	4.09	4.09	4.090

**Table 2:** The coating conditions of G1, G2, G3, and G4.

Coating	Constitute
G1	EP coating
G2	EP coating + corrosion inhibitor
G3	CeO_2_-GO(4:1)/EP coating
G4	CeO_2_-GO(4:1)/EP coating + corrosion inhibitor

**Table 3:** Each coating’s carrier density for 15 d, 30 d, 45 d, 60 d in 20% Seawater.

Coating	15 d N_D_ (cm^−3^)	30 d N_D_ (cm^−3^)	45 d N_D_ (cm^−3^)	60 d N_D_ (cm^−3^)
G1	4.681 × 10^13^	2.235 × 10^14^	3.973 × 10^13^	7.123 × 10^14^
G2	1.540 × 10^13^	1.837 × 10^14^	3.620 × 10^13^	2.008 × 10^14^
G3	2.645 × 10^13^	1.708 × 10^12^	9.865 × 10^12^	5.399 × 10^12^
G4	2.163 × 10^11^	1.101 × 10^12^	6.374 × 10^12^	2.202 × 10^12^

**Table 4:** Each coating’s carrier density for 15 d, 30 d, 45 d, 60 d in 40% Seawater.

Coating	15 d N_D_ (cm^−3^)	30 d N_D_ (cm^−3^)	45 d N_D_ (cm^−3^)	60 d N_D_ (cm^−3^)
G1	7.331 × 10^14^	2.790 × 10^15^	2.871 × 10^15^	9.372 × 10^15^
G2	4.542 × 10^14^	8.691 × 10^14^	6.042 × 10^14^	2.362 × 10^15^
G3	3.101 × 10^12^	2.646 × 10^13^	2.115 × 10^13^	1.997 × 10^14^
G4	6.937 × 10^11^	3.207 × 10^12^	3.417 × 10^12^	4.112 × 10^12^

**Table 5:** Each coating’s carrier density for 15 d, 30 d, 45 d, 60 d in 60% Seawater.

Coating	15 d N_D_ (cm^−3^)	30 d N_D_ (cm^−3^)	45 d N_D_ (cm^−3^)	60 d N_D_ (cm^−3^)
G1	4.091 × 10^15^	3.960 × 10^16^	2.470 × 10^16^	2.563 × 10^17^
G2	3.340 × 10^15^	2.564 × 10^16^	2.280 × 10^16^	1.303 × 10^17^
G3	3.715 × 10^13^	6.830 × 10^14^	8.861 × 10^15^	3.483 × 10^16^
G4	2.663 × 10^13^	5.728 × 10^14^	1.266 × 10^13^	1.040 × 10^16^

**Table 6:** Kinetic parameters of each coating in seawater of 20% concentration for 90 days.

Coating	E_corr_ (V)	I_corr_ (A/cm^2^)	η (%)	R_p_ (ohm)
G1	−0.866	2.473 × 10^−6^	/	4.395 × 10^5^
G2	−0.723	4.781 × 10^−^^7^	80.67	2.564 × 10^6^
G3	−0.627	2.804 × 10^−8^	98.87	4.214 × 10^7^
G4	−0.487	1.543 × 10^−^^9^	99.93	7.170 × 10^8^

**Table 7:** Kinetic parameters of each coating in seawater of 40% concentration for 90 days.

Coating	E_corr_ (V)	I_corr_ (A/cm^2^)	η (%)	R_p_ (ohm)
G1	−0.981	3.963 × 10^−6^	/	2.858 × 10^5^
G2	−0.731	1.003 × 10^−6^	72.77	4.650 × 10^5^
G3	−0.661	1.591 × 10^−7^	95.69	4.370 × 10^6^
G4	−0.526	8.249 × 10^−^^8^	97.77	6.733 × 10^6^

**Table 8:** Kinetic parameters of each coating in seawater of 40% concentration for 90 days.

Coating	E_corr_ (V)	I_corr_ (A/cm^2^)	η (%)	R_p_ (ohm)
G1	−0.916	4.274 × 10^−^^6^	/	2.687 × 10^5^
G2	−0.782	1.417 × 10^−^^6^	66.81	3.755 × 10^5^
G3	−0.695	7.903 × 10^−6^	86.75	7.918 × 10^5^
G4	−0.658	3.581 × 10^−^^7^	91.62	1.162 × 10^6^

## Data Availability

Not applicable.
